# Scleredema in the Setting of Monoclonal Gammopathy of Unknown Significance With Progression to Multiple Myeloma: A Case Report

**DOI:** 10.7759/cureus.44968

**Published:** 2023-09-09

**Authors:** Austin L Nickell, Megan Corn, Devendranath Mannuru, Alicia M Hinze

**Affiliations:** 1 Internal Medicine, University of North Dakota School of Medicine and Health Sciences, Grand Forks, USA; 2 Obstetrics and Gynaecology, University of North Dakota School of Medicine and Health Sciences, Grand Forks, USA; 3 Internal Medicine, University of North Dakota School of Medicine and Health Sciences, Fargo, USA; 4 Rheumatology, Mayo Clinic, Rochester, USA

**Keywords:** plasmacytoma, organomegaly, scleroderma, raynaud’s phenomenon, multiple myeloma, monoclonal gammopathy of undetermined significance (mgus)

## Abstract

Type 2 scleredema on the background of monoclonal gammopathy of undetermined significance (MGUS) is a rare and progressive connective tissue disorder with very few cases reported to date. It is characterized by chronic and diffuse induration of the skin that begins in the upper back and neck and progresses proximally to distally, involving the shoulders, trunk, and arms; the hands are usually spared. Here, we present an unusual case of long-standing scleredema that progressed to involve the hands and fingers. This case was further complicated by new-onset Raynaud’s phenomenon, splenomegaly, lymphadenopathy, the development of a plasmacytoma, and eventual progression to multiple myeloma. We highlight the differential diagnoses for his complex presentation, the workup that was completed, and current treatment options.

## Introduction

Scleredema is a rare and progressive connective tissue disorder characterized by chronic and diffuse induration of the skin [1]. A physical examination demonstrates non-pitting edema. The typical course begins at the upper back and neck and progresses proximally to distally, involving the shoulders, trunk, and arms [1]. The hands and feet are usually spared [1]. Histologically, the dermis reveals thickened collagen bundles separated by clear mucin-filled spaces [2].

Scleredema has been classified into three types based on etiology [2]. Type 1 scleredema presents two to three weeks following a febrile illness [2]. Type 2 is associated with hematological findings including multiple myeloma and monoclonal gammopathy of undetermined significance (MGUS) [2]. Type 3 is associated with severe and poorly managed type I diabetes mellitus [2]. To the best of our knowledge, under 50 cases of scleredema related to monoclonal gammopathy of undetermined significance (MGUS) have been reported in the literature to date, and even fewer cases of scleredema have been associated with multiple myeloma.

There is no specific treatment for scleredema, although steroids, immunosuppression, phototherapy, and intravenous immunoglobulin have been used with varying success [2-4]. For scleredema associated with MGUS or multiple myeloma, systemic chemotherapy has been shown in case reports to reduce symptom severity [5, 6].

The prognosis of scleredema varies based on subtype [2]. Type 1 scleredema is usually self-limiting and resolves within six to 24 months [2]. Type 2 and 3 progress slowly and may be accompanied by myelodysplastic syndrome, diabetic vascular complications, and pleural or pericardial effusions [2]. 

This case report was previously presented at the University of North Dakota's Frank Low Research Day on April 12, 2023.

## Case presentation

A middle-aged man with a three-year history of MGUS presented to the clinic with a history of slowly progressive skin tightening and thickening, the recent onset of Raynaud’s phenomenon without digital ulcers, dysphagia with progressive choking of liquids and solids, gastroesophageal reflux disease (GERD), dyspnea, and numbness and tingling in the feet. The cutaneous symptoms began 10 years prior to his initial presentation with skin thickness over his neck and chest, which progressed slowly to involve his shoulders, upper back, torso, and upper arms. His skin progressively hardened over time; he described early and significant limitations in the range of motion at his shoulders. He had previously been diagnosed with scleroderma, per his recollection at the onset of symptoms, and had taken methotrexate, prednisone, and pentoxifylline. He did not note any benefit from these medications and was eventually lost to follow-up. His condition continued to progress, with thickening over his forearms and fingers over the last one to two years.

**Figure 1:**
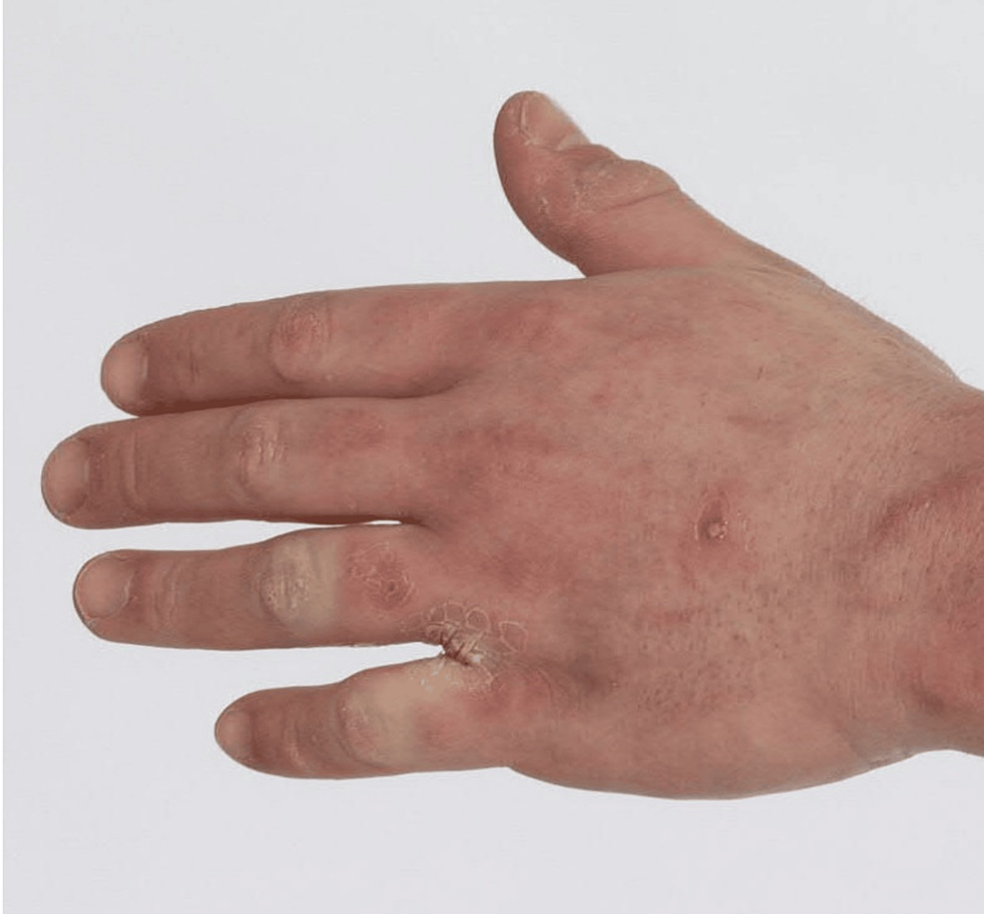
Image of left hand showing thickness of the skin over the fingers, giving the appearance of sclerodactyly.

On exam, he was found to have thick skin over the thorax, face, upper back, upper arms, forearms, and fingers, with proximal sites thicker than distal sites (Figure 1). The lower extremities were unaffected. There were no areas of cutaneous atrophy or bound-down skin, and there were no pigment changes. There were no waxy papules on the posterior auricular areas, forehead, glabella, posterior neck, or fingers. There were no telangiectasias or calcinosis. A video nailfold capillaroscopy showed peri-capillary edema but did not show other abnormal features of capillary dilatation or nailfold capillary drop-out. Antinuclear antibody (ANA) by human epithelial (HEp2) cells, anti-scleroderma 70 antibody, anti-centromere antibody, and ribonucleic acid polymerase III antibody labs were negative. The thyroid stimulating hormone (TSH) and vascular endothelial growth factor (VEGF) levels were normal. Biopsies of his neck, chest, and forearm showed pan-dermal sclerosis with focal increased interstitial mucin, consistent with scleredema.

Evaluation of his other presenting symptoms included a pulmonary function test to work up his dyspnea, which was consistent with a restrictive physiology. A chest CT showed clear lungs without evidence of edema, ground-glass opacities, infiltrative processes, or interstitial lung disease. An echocardiogram showed a left ventricular ejection fraction of 55% and increased left ventricle wall thickness. An amyloid scan ruled out transthyretin amyloidosis. A right and left cardiac catheterization revealed normal cardiac hemodynamics at rest and exercise, without evidence of cardiac output limitation or evidence of right or left heart failure. There was no atherosclerotic disease of the coronary arteries. Dyspnea was ultimately attributed to restrictive lung physiology, with contributions thought to be multifactorial and secondary to cutaneous disease of the thorax and the patient’s weight (BMI 37 kg/m2). A barium esophagram to work up his progressive choking of liquids and solids noted normal pharyngeal function, normal peristaltic waves, and no strictures. Esophagogastroduodenoscopy was recommended but not performed at that time. Electromyography (EMG) was ordered to investigate the sensory changes in the feet but was ultimately not performed.

The patient had a previous diagnosis of MGUS with 0.4 g/dL immunoglobulin G (IgG) kappa monoclonal proteins and a kappa light chain-restricted plasma cell dyscrasia occupying 5% of his bone marrow. Repeat hematologic testing revealed an M-spike increase to 0.8 g/dL IgG kappa with 5% to 9% plasma cells in the bone marrow. Renal function tests were normal, and no light chains were detected in his urine. Hemoglobin and serum calcium were also normal. A bone survey showed no osseous abnormality with the exception of a small, non-specific oval lucency at the vertex; a follow-up CT of the head did not identify a lesion. He was offered treatment designed to eliminate the plasma cell clone, although the impact on scleredema could not be guaranteed. He opted not to pursue treatment.

**Figure 2:**
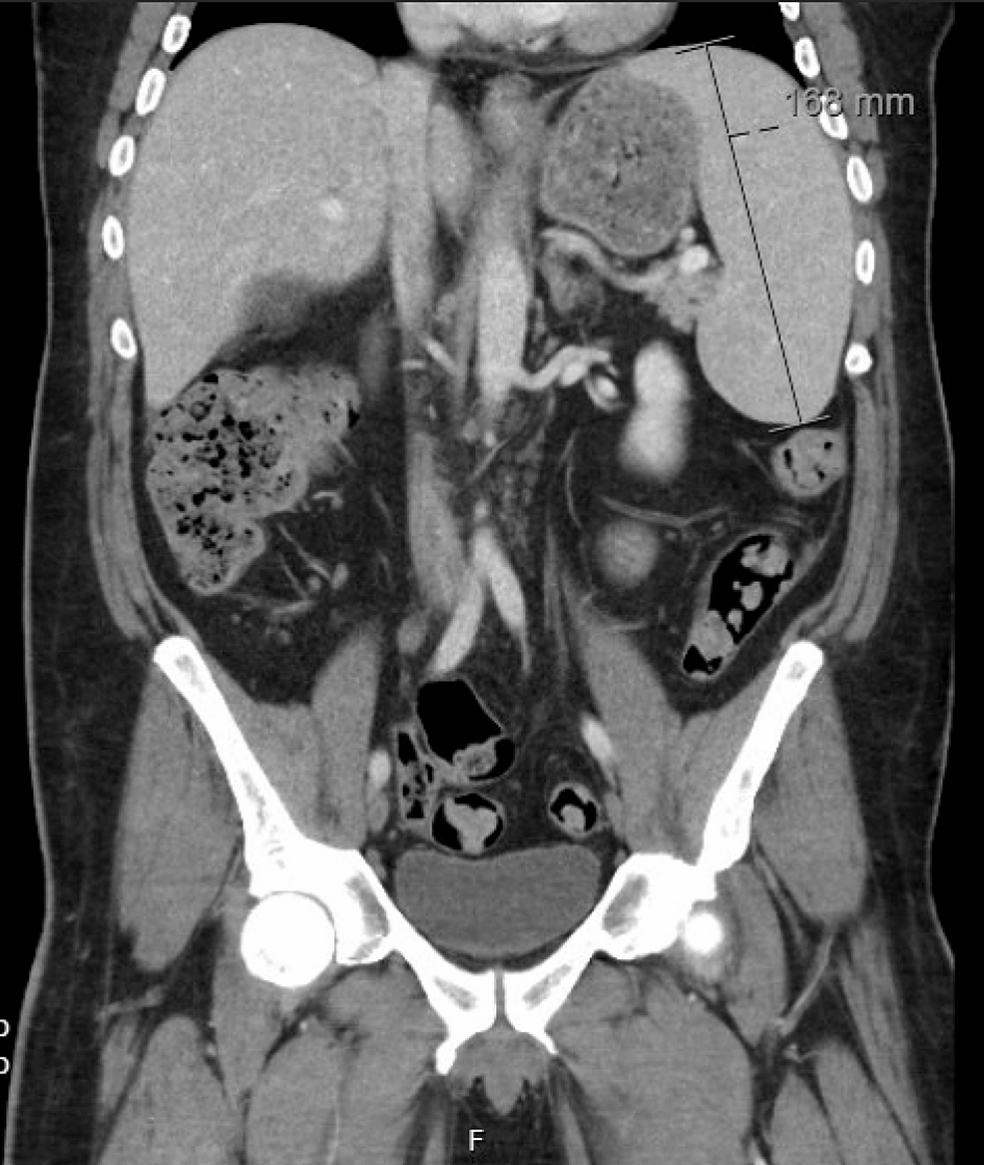
CT of the abdomen and pelvis showing an enlarged spleen measuring 16.1 cm in greatest dimension

**Figure 3:**
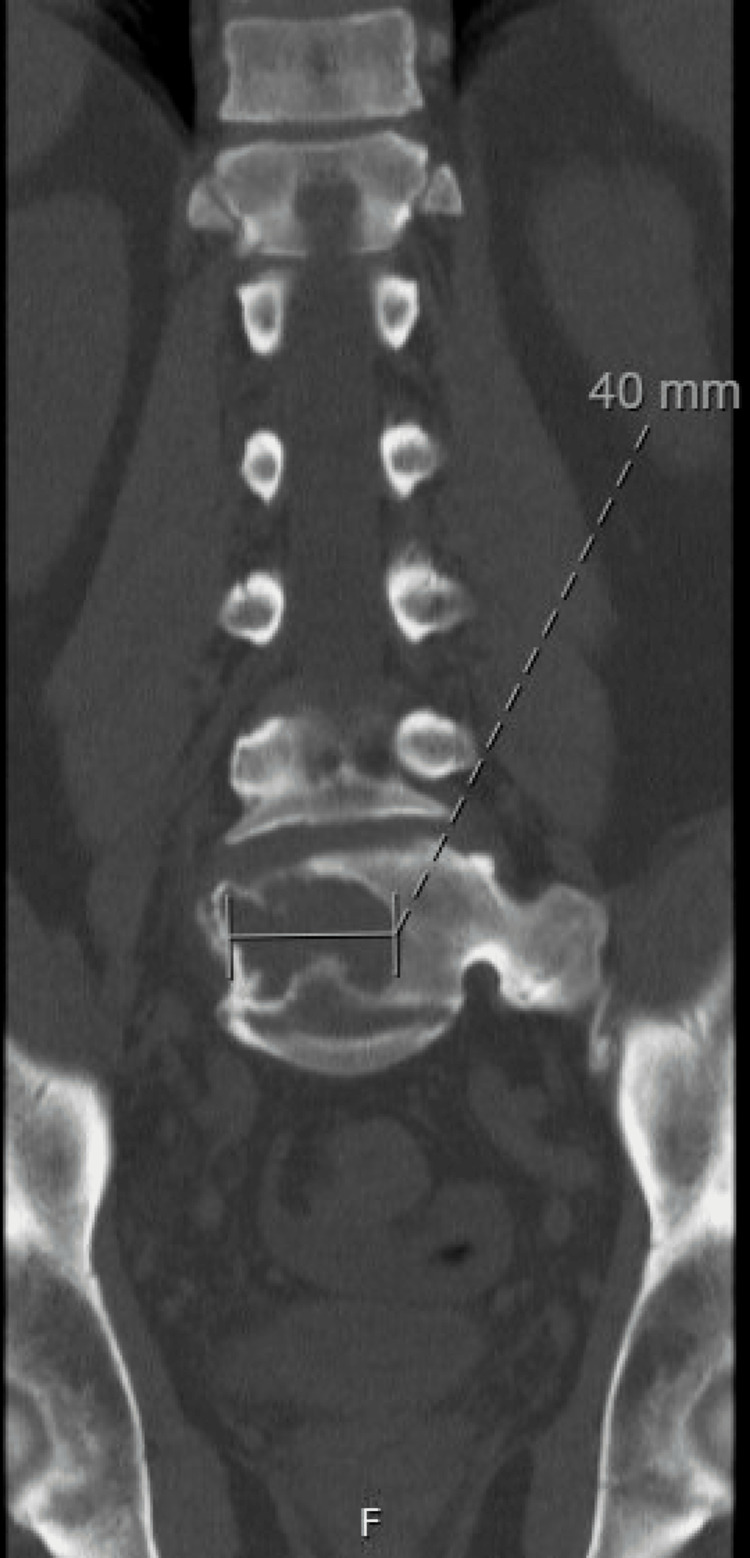
CT of the lumbar spine demonstrating a lytic lesion within the L5 vertebra measuring 40 mm in greatest dimension

**Figure 4:**
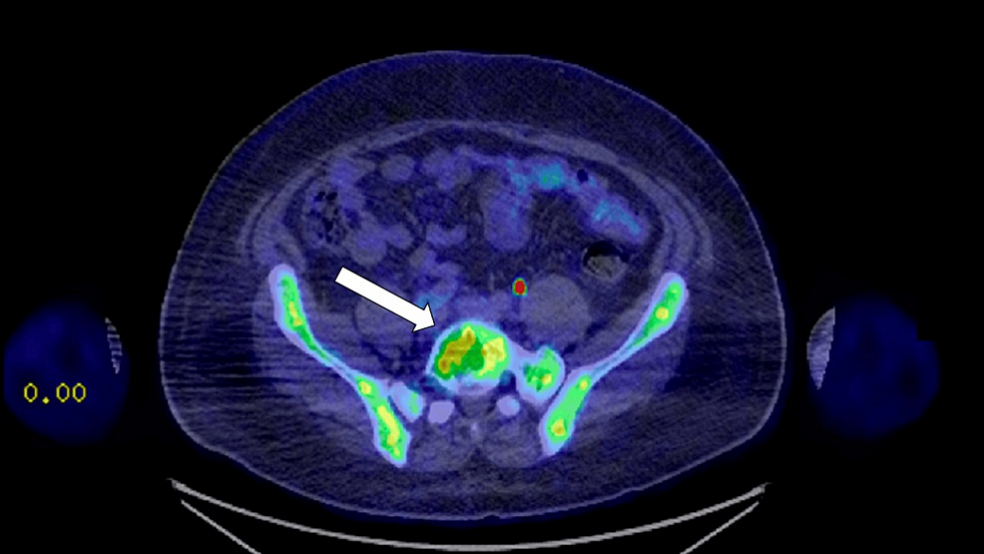
Axial PET-CT image showing FDG avidity of the lytic lesion in the L5 vertebral body (solid white arrow) PET-CT: Positron emission tomography-computed tomography, FDG: F-fluorodeoxyglucose

**Figure 5:**
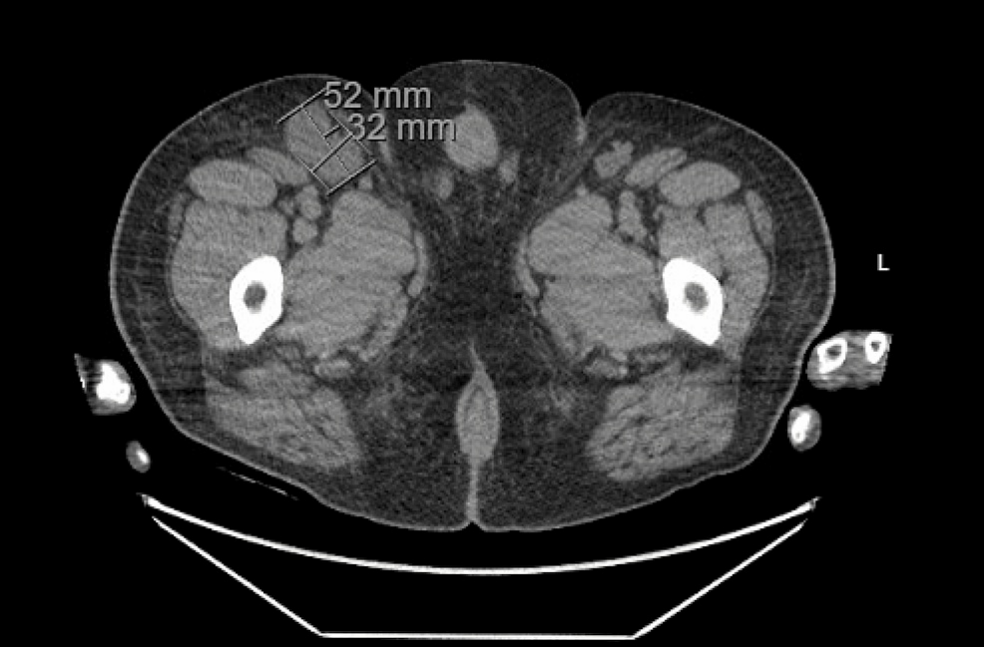
CT of the abdomen and pelvis in axial view demonstrating an inguinal nodal conglomerate measuring 5.2 cm x 3.2 cm

Two years following his initial presentation, the patient presented to internal medicine with a weight loss of 60 pounds and associated abdominal pain, nausea, vomiting, and right-sided lumbar spinal pain. Imaging revealed a 16.8 cm enlarged spleen (Figure 2), mild gastric wall thickening, circumferential thickening of the proximal small bowel, and a lytic lesion in the central aspect of the L5 vertebral body (Figure 3). Positron emission tomography and computed tomography (PET-CT) showed F-fluorodeoxyglucose (FDG) avidity of the lytic L5 vertebral body lesion with a standardized uptake value (SUV) max of 5.5 cm (Figure 4). No other lytic lesions were identified. A biopsy of the L5 lesion was completed and was consistent with a plasmacytoma. Immunohistological staining showed CD138-positive plasma cells aberrantly expressing cyclin D1 and CD56; they were negative for CD20. The cells were monoclonal kappa light chain restricted by kappa and lambda in situ hybridization. The PET-CT scan additionally revealed a right inguinal nodal conglomerate measuring 5.2 cm x 3.2 cm along with external iliac and pelvic sidewall adenopathy (Figure 5).

The patient underwent a biopsy of the right inguinal mass, which was negative for infectious or malignant causes and was deemed a reactive process. The patient did not note further progression or worsening of his skin thickening during this time and did not elect to follow up with rheumatology or dermatology for a repeat assessment. Lab results at the time of the plasmacytoma diagnosis included a serum calcium level of 9.3 mg/dL, hemoglobin of 16.3 g/dL, and an estimated creatinine clearance of 195.4 mL/min. Thus, the patient did not have evidence of end-organ damage attributable to the underlying plasma cell disorder and did not meet the criteria for multiple myeloma at this time according to the revised International Myeloma Working Group Criteria for the diagnosis of multiple myeloma [7].

**Figure 6:**
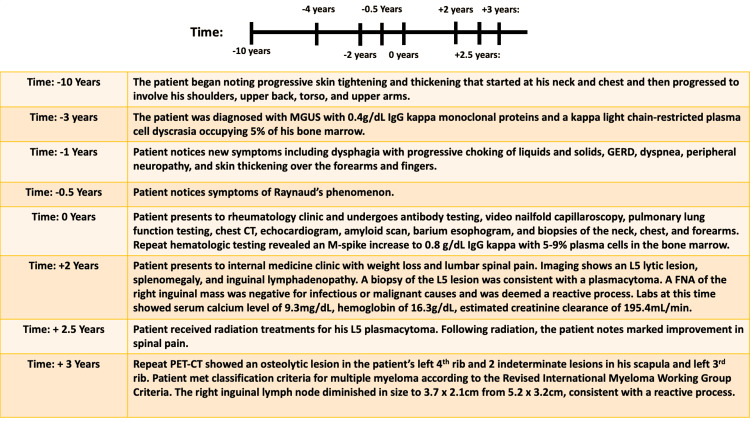
Timeline of patient’s disease course MGUS: Monoclonal gammopathy of undetermined significance, IgG: Immunoglobulin G, GERD: Gastroesophageal reflux disease, FNA: Fine-needle aspiration, PET-CT: Positron emission tomography-computed tomography

Radiation to the L5 plasmacytoma was pursued. In total, the patient received 3750 cGy in 23 fractions, with 250 cGy per fraction, using image-guided radiation and 3D conformal radiation therapy. Following radiation treatments, the patient noted a marked improvement in spinal pain. A timeline depicting the patient’s clinical course is described in Figure 6.

A repeat PET-CT was obtained in the context of recurrent back pain seven months after his initial PET-CT. This showed an osteolytic lesion (left 4th rib, 15.1 mm in greatest dimension, SUV max 2.8) and two indeterminate lesions (scapula, 10 mm in greatest dimension, SUV max 2.1; left 3rd rib, 7.2mm, SUV max 1.5). With the history of the plasmacytoma and the single lytic rib lesion, along with two further indeterminate lesions, it was felt he met the classification criteria for multiple myeloma according to the revised International Myeloma Working Group Criteria [7]. Notably, the right inguinal lymph node diminished in size to 3.7 cm x 2.1 cm from 5.2 cm x 3.2 cm, most consistent with a reactive process. Due to the patient’s scleredema and multiple myeloma, systemic chemotherapy was again discussed but ultimately delayed in the setting of acute cholecystitis and the need for cholecystectomy. Furthermore, the patient suffered postoperative complications, including a hematoma, cellulitis, deep vein thrombosis (DVT), delayed wound healing, and *Clostridium difficile* colitis.

The patient is set to receive four to six 28-day cycles of chemotherapy consisting of daratumumab and hyaluronidase-fihj and lenalidomide. Dexamethasone will also be given prior to daratumumab and hyaluronidase-fihj for hypersensitivity reaction prophylaxis. Cycle one will consist of daratumumab and hyaluronidase-fihj 1800 mg subcutaneous injections with 20 mg of dexamethasone on days 1, 8, 15, and 22. He will then begin taking lenalidomide 25 mg capsules daily starting on the second cycle. The main side effects anticipated with these chemotherapeutic agents include myelosuppression, increased risk of DVT, hypersensitivity reactions, and injection site reactions.

## Discussion

This patient presented with an unusual course of scleredema in that there was additional cutaneous thickening and edema of his hands and fingers, along with Raynaud’s phenomenon. The involvement of his hands in combination with Raynaud’s phenomenon raised the question of concurrent systemic sclerosis in the background of scleredema. However, cutaneous changes progressed from proximal to distal; cutaneous changes secondary to systemic sclerosis progressed from distal to proximal. In Raynaud's disease associated with systemic sclerosis, nailfold capillary abnormalities are seen with a prevalence near 80% [8]. Our patient’s nailfold capillaries were largely normal. His serological evaluation was negative; he had no other cutaneous stigmata, such as telangiectasias and calcinosis, and no evidence of interstitial lung disease or pulmonary arterial hypertension to suggest systemic sclerosis. Histopathology from a cutaneous biopsy was consistent with scleredema with increased intradermal mucin. Scleromyxedema was considered in the differential, which is also associated with monoclonal gammopathy and increased intradermal mucin on histopathology [9]. Extracutaneous features may include Raynaud’s phenomenon, gastrointestinal signs and symptoms including dysphagia and objective dysmotility, and pulmonary symptoms with restrictive or obstructive ventilatory defects [10]. Cutaneous features of scleromyxedema include waxy papules, typically located on the posterior-auricular area, forehead, glabella, posterior neck, and fingers; these characteristic waxy papules were not seen on this patient’s cutaneous exam. Polyneuropathy, organomegaly, endocrinopathy, monoclonal gammopathy, and skin abnormalities (POEMS) were considered as sclerodermatous skin changes and Raynaud’s phenomenon have been described and would satisfy one minor criterion. However, 95% of POEMS cases have a lambda-restricted light chain monoclonal gammopathy, and our patient had a kappa restriction [11]. Furthermore, normal plasma VEGF is rarely observed in POEMS [12]. With the significant inguinal lymphadenopathy, Castleman disease, which can be seen in POEMS, was a consideration. However, the regression in lymph node size over time suggests a reactive process and would be unexpected in Castleman disease in the absence of treatment. The patient did not have other features such as endocrinopathy, extracellular volume overload, thrombocytosis, or polycythemia, making POEMS less likely [13-15]. We were unable to fully characterize the sensory changes in the feet as the patient did not complete an EMG; in POEMS, demyelinating polyneuropathy is most common and usually progressive [14]. Symptomatic progressive neuropathy had not occurred at the time of the evaluation for plasmacytoma.

Other differentials for late-onset (age over 30) Raynaud’s phenomenon can include systemic lupus erythematosus (SLE), Sjögren’s disease, idiopathic inflammatory myopathies, cryoglobulinemia, paraproteinemia, pheochromocytomas, carcinoid syndrome, thyroid disease, or vasospasm secondary to pharmaceuticals (ergots, methysergide, beta-blockers, dextroamphetamine, methylphenidate, bleomycin, cisplatin, clonidine, cocaine, cyclosporine, interferon-alpha, and nicotine) [16]. While cryoglobulinemia is possible in the setting of MGUS, this laboratory test was unable to be obtained. He had no other features of cryoglobulinemia syndrome, such as palpable purpura or renal disease. Other than paraproteinemia, our patient did not have findings specific to the other differentials.

The pathogenesis of scleredema is not well understood but is likely due to increased fibroblast production of type 1 collagen and glycosaminoglycans in the reticular dermis, as well as mucopolysaccharide production in the interfibrillar dermal space [16,17,2]. Keragala et al. hypothesized that paraproteins in MGUS act like antibodies, directly stimulating fibroblasts [6].

Several systemic treatments have been trialed for scleredema, including corticosteroids, pentoxifylline, cyclosporine, methotrexate, hydroxychloroquine, and intravenous immunoglobulin; however, none are universally accepted and are often not successful [16]. In scleredema with underlying MGUS, chemotherapeutic regimens have resulted in clinical improvement in some cases [5,6]. Chemotherapeutic agents that have been used include cyclophosphamide, vincristine, melphalan, thalidomide, and bortezomib [6,18]. Phototherapy with UVA1 has shown positive results in some patients [14,19]. Physiotherapy is usually implemented in an effort to improve or maintain range of motion.

## Conclusions

Scleredema is a rare and progressive connective tissue disorder that has a poorly understood pathogenesis. It can occur following a febrile illness or in association with type II diabetes, MGUS, or multiple myeloma. The disease's insidious onset can hinder a timely diagnosis, and its symptoms can readily be mistaken for other sclerosing skin conditions. Differential diagnoses for the disease include systemic sclerosis, scleromyxedema, and POEMS. Features that help distinguish scleredema from other sclerosing skin conditions include the location of skin involvement and the pattern of progression. While an overlap of limited cutaneous systemic sclerosis cannot be entirely excluded in this patient with the involvement of his hands and fingers, the negative serological testing, normal nailfold capillaries, absence of other cutaneous stigmata, including telangiectasias and calcinosis, absence of interstitial lung disease, and absence of objective signs of esophageal dysmotility by barium esophagram make this less likely. Therefore, an atypical presentation of scleredema with involvement of the hands, rather than the manifestation of two rare diseases, is favored. It is possible that the long duration (over 10 years) resulted in more extensive cutaneous involvement than previously reported in type 2 scleredema. Scleredema with MGUS or multiple myeloma has been shown in case studies to improve with systemic chemotherapy, notably with cyclophosphamide, vincristine, melphalan, thalidomide, or bortezomib.
